# Selenium Nanoparticles Ameliorate Adverse Impacts of Aflatoxin in Nile Tilapia with Special Reference to *Streptococcus agalactiae* Infection

**DOI:** 10.1007/s12011-023-04031-1

**Published:** 2023-12-26

**Authors:** Ahmed H. Sherif, Mohsen A. Zommara

**Affiliations:** 1 Fish Diseases Department, Animal Health Research Institute, Agriculture Research Center (ARC), Kafrelsheikh, 12619 Egypt; 2 Dairy Sciences Department, Faculty of Agriculture, Kafrelsheikh University, Kafrelsheikh, 33511 Egypt

**Keywords:** *Oreochromis niloticus*, Selenium nanoparticles, Aflatoxin, Cytokine, Antioxidants, Innate immunity, DNA fragmentation

## Abstract

Aflatoxin B1 (AFB1) is a plant-origin toxin that could induce oxidative stress in fish. The micromineral selenium (Se) possesses well-documented antioxidant properties. To assess the ameliorative effects of SeNPs (1 mg/kg fish feed) on oxidative stress induced by AFB1 (500 μg/kg fish feed), Nile tilapia (32.2±1.7 g body weight) were distributed randomly and even in six groups for 8-week feeding trial. Live enzymes, AST, ALT, and ALP levels were increased in the serum of fish fed AFB1-contaminated diet, and the addition of SeNPs could restore normal values compared to the control. The gene expression of antioxidant enzymes, superoxide dismutase (SOD) enzyme and catalase (CAT) enzyme, and DNA fragmentation were significantly increased in response to aflatoxin exposure, while dietary SeNPs could mitigate the generated oxidative stress. The innate immunity, serum antibacterial activity (SAA), oxidative burst activity (OBA), phagocytic activities (PA and PI), and gene expression of cytokines (interleukin (*IL*)*-1β*, heat shock protein70 (*Hsp*), and tumor necrosis factor (*TNF*)-*α*) revealed a status of immunosuppression in Nile tilapia fed on AFB1-contaminated diet. These findings showed that fish became more vulnerable to *Streptococcus agalactiae* infection with a high mortality rate while dietary SeNPs provided a high relative protection level (RPL). From the obtained findings, SeNPs could mitigate the oxidative stress induced by feeding the AFB1 diet and could boost the immunity of stressed Nile tilapia.

**Supplementary Information:**

The online version contains supplementary material available at 10.1007/s12011-023-04031-1.

## Introduction

Tilapia is currently among the most widely farmed fish species in the world, second only to carp, with production of over 6 million mt in 2019, valued at over 12 billion US$ [1]. Wherein Egypt, Nile tilapia fish *(Oreochromis niloticus)* is the most preferred cultured fish species by Egyptian consumers due to its high palatability and convenient price [2]. The high production of freshwater fish farms places Egypt in the second rank following China all over the world [3].

Additionally, high price fish meal is a common source of protein in most aquafeeds [4]; therefore, replacement of fish meal with cereal crops in aquatic diet formulation is necessary, and unfortunately, plant-based diets contain various antinutrients, such as phytate, which are chelated with trace minerals, rendering them unavailable [5, 6]. So, supplemental essential trace minerals are necessary for fish feeds at optimum levels [7]. Microminerals such as selenium (Se), manganese (Mn), zinc (Zn), copper (Cu), iodine (I), and iron (Fe) play different roles in the fish body, including the development and homeostasis of the skeletal system; composition of organic molecules such as proteins, lipids, and hormones; enhancement of enzymatic activities; and maintenance of the osmotic balance [6–8]. Several studies investigate the use of nanosized minerals to combat bacterial infection in Nile tilapia such as Se, titanium dioxide nanoparticles (anatase and rutile crystal) and zinc nanoparticles [9–14], and natural products such as *Moringa oleifera* [15], *Nigella sativa* oil [14], and silymarin [16]. The micromineral (Se) possesses an essential metabolic role saving the antioxidant status in the animal body [17, 18]. Meanwhile, Se is an essential micromineral in animal feed with a narrow therapeutic index against immunotoxicity [19]. Currently, several studies stated that nanometals could be used in fish feed to fulfill their dietary requirements, and SeNPs boost immune and antioxidant status along with high bioavailability and lower toxicity in Nile tilapia [2], common carp [20], and rainbow trout [21]. Recently, organic nanomaterials have been preferred, and Se-enriched yeast (Se-yeast) has become the main used form [22]. The nutritional requirements of selenium were estimated in different fish species: 1.06–2.06 mg/kg for Nile tilapia [23] and 0.73–1.19 mg/kg for gibel carp (*Carassius auratus gibelio* var. CAS III) [24].

Nile tilapia that received SeNPs at a level of 2 mg/kg fish feed showed significant enhancements in growth performance and survival rate [13]. Dietary selenium yeast at the rate of 3.3 mg/kg diet (2.36 mg selenomethionine and 0.94 mg organic selenium) for 60 days could protect Nile tilapia against chronic toxicity of glyphosate and/or malathion via mitigating the generated oxidative inflammation [11]. Naturally, selenium presents in organic forms as selenomethionine and selena-cysteine [25] and inorganic elemental state (Se0) as selenides (Se2−), selenates, or selenites [26]. Many factors affect the selenium transformation pH, amount of free oxygen, redox potential, and humidity [27].

Many authors suggested different dietary supplementation rates of Se, Se-enriched yeast 0.5–4 g/kg [11], animal feeds of 0.5 mg/kg [28], up to 0.2 mg/kg (1–1.1 mg/kg assessed dietary selenium) in juvenile gilthead seabream [29].

The extensive use of cereal ingredients in feed formulations may lead to an increase in the occurrence of aflatoxicosis, mainly with improper storage [30, 31]. Hence, Nile tilapia is by far the most cultivated fish species in Egypt and is highly AFB1 sensitive [32]. Feeding AFB1-contaminated feed resulted in generated oxidative stress, which led to immunotoxicity, inflammation reaction, and apoptosis in animals [33, 34].

Selenium is an antioxidant agent that induces the production of glutathione peroxidase (GPx) to counteract oxidative stress [35]. Se deficiency, fish became more vulnerable to microbial infection and sensitivity to AFB1-induced toxicity in the spleen, which is one of the lymphoid organs responsible for humoral and cellular immune responses [36, 37]. Detoxification of AFB1 by physical and chemical methods has several drawbacks, such as loss of some nutrients and decreased palatability. Moreover, the equipment required for the application of these techniques is too expensive for farmers [38].

So, this work investigates the oxidative stress in Nile tilapia that resulted from feeding on AFB1-contaminated diet. Also, the potential ameliorating role of nanoselenium SeNPs was evaluated against aflatoxin (AFB1) stress.

## Materials and Methods

### Fish Accommodation and Trial Design

Experimental Nile tilapia (*Oreochromis niloticus*), with an average weight of 32.2 ± 1.7 g, were purchased from a local private freshwater fish farm at Um-sin village in Kafrelsheikh, Egypt. Nile tilapia was transported to wet the laboratory of the Animal Health Research Institute. According to [39–41], fish were tranquilized at the farm with MS222 (SyncaineR, Syndel, Canada) at a dose of 40 mg/L. On arrival, the fish were disinfected with a bath of iodine (20 ppm/L), the trade name is Betadine® containing 5% povidone-iodine, the product was purchased from the local market and manufactured by Nile Company for Pharmaceuticals, Egypt, and then fish were stocked in fiberglass tank 1.5 × 1 m. Water quality was checked day after day.

In the wet laboratory, fish were acclimatized for 14 days at experimental conditions: water temperature, pH, and salinity were 27.5 ± 0.5 °C, 7.9 ± 0.1, and 0.48 ± 0.1 g/L, respectively. Day after day, only one-third of tank water was exchanged with clean un-chlorinated water to maintain constantly suitable water parameters.

Fish feed was offered at 0.9.00 a.m. and 03.30 p.m. at a rate of 5% b.w. fish per day, basal diet composition: crude protein 30%, digestible energy 4000 kcal/kg. The diet was purchased from the local market and manufactured by Aller Aqua® Egypt (Reg. no. 9782). Lot. no. 365622 https://www.aller-aqua.com/.

Fish were subdivided into five groups and fed on diets supplemented with SeNPs and AFB1 at a concentration of 1 mg/kg [2] and 500 μg/kg fish feed [37], respectively, following:

- **G1:** Fish fed the basal diet are considered as a control group.
- **G2:** Fish fed the basal diet supplied with SeNPs for 4 weeks.
- **G3:** Fish fed an AFB1-contaminated diet for 4 weeks.
- **G4:** Fish fed an AFB1-contaminated diet plus SeNPs for 4 weeks.
- **G5:** Fish fed an AFB1-contaminated for 4 weeks then SeNPs with the basal diet for another 4 weeks.
- **G6:** Fish fed an AFB1-contaminated diet for 4 weeks then SeNPs plus AFB1-contaminated diet for another 4 weeks.

### Additive Sources and Preparation

#### Aflatoxin (AFB1) Preparation

Following the method of Abdelhamid and Mahmoud [42], corn pellets were fermented using *Aspergillus parasiticus* (NRRL 2999) which was grown in synthetic media, yeast extract-sucrose broth containing 2% yeast extract and 20% sucrose. The substrate was dispensed in conical flasks. The flasks were then autoclaved for 15 min at 121 °C, then cooled and inoculated with spore suspension and incubated for 9 days at 25–29 °C then AFB1 concentration was determined using quantitative thin layer chromatography TLC [43].

#### Preparation of SeNPs and Diet Incorporation

The SeNPs were manufactured following the methods described by Zommara [44] and Prokisch et al. [45] using lactic acid bacteria (LAB-Se, Lactomicrosel®). Briefly, SeNPs were manufactured using pure yogurt bacterial cultures containing *Lactobacillus delbrueckii* subsp. *bulgaricus* (NCAIM B 02206) and *Streptococcus thermophilus* (CNCM I-1670). The size of the obtained SeNPs was determined and photographed using a scanning electron microscope (SEM) (JSM-IT100, JEOL Co. Japan) photos of the cultured media after 72 h [46, 47]. The manufactured SeNPs were uniformly distributed in Milli-Q water (1 mg/mL) using an ultrasonic [48]. Thereafter, fish food (pellet form) was soaked and fully homogenated until paste formation. Then, the gelatine was added to feed past/Se NP mixture to improve feed consistency (Canal Aqua Cure, Egypt) and left to dry at room temperature then was evenly cut into small sizes.

### Liver Enzymes

The levels of the liver enzymes in the serum of the experimental fish, aspartate amino transaminase (AST) and alanine amino transaminase (ALT), were colometerically detected to evaluate the effect of AFB1 and the ameliorative role of SeNPs according to the methods reported by Reitman and Frankel [49]. AST used aspartate and 2-oxoglutarate to obtain glutamate and oxalacetate and ALT used alanine and 2-oxoglutrate and obtain glutamate and pyruvate. The oxalacetate or pyruvate formed in the above reaction reacts with 2-4-dinitrophenyl hydrazine to form phenyl hydrazone. The end compound generates a color that could be measured at the wavelength of 546 nm. The color intensity is related to enzyme activity compared to a standard reference. Levels of alkaline phosphatase (ALP) activity were detected according to methods mentioned by Rec [50]. All kits and reagents were provided by Spectrum Diagnostic Co.

### Innate Immunity

#### Serum Antibacterial Activity (SAA)

The activity of Nile tilapia serum was tested against bacterial in fish that received dietary-active-MOS and the control [51]; briefly, *O. niloticus* serum and a bacterial suspension of *A. hydrophila* (2 × 10^8^ CFU) were combined in equal quantities (100 μL) and incubated at 25 °C for 1 h. Furthermore, the blank (control) was formed with sterile PBS instead of fish serum. A dilution at 1:10 with sterile PBS of 100 μL of the serum-bacterial mixture was placed on blood agar and placed in an incubator (27 °C/24 h). The colonies that grew on the tryptic soy agar were counted as viable bacteria.

#### Oxidative Burst Activity (OBA)

According to Anderson et al. [52], the OBA of the experimental fish heterophils was determined using a nitroblue tetrazolium assay briefly; within 15 min following the blood sample collection, a drop of heparinized fish blood was put on a coverslip then the coverslips were incubated in humid chambers for 30 min at room temperature (25 °C), and the neutrophils adhere to the coverslip, which was gently washed with PBS (pH 7.4). The coverslip was stained with NBT 0.2% solution. The stain was manufactured by Fluka Buchs, Switzerland. After 30 min of incubation, the dark blue-stained cells were considered positive and counted under a light microscope.

#### Phagocytosis Activity

Following the method of Kawahara et al. [53], heterophiles were isolated [54], then a 24-h-old of culture *Candida albicans* with a dose of 1 × 10^6^ cells/mL, and heterophile count was adjusted at 2.5 × 10^6^ viable cells/mL, and a mixture of 1 mL of the *C*. *albicans* suspension and 1 mL of collected heterophiles was incubated 27 °C/1 h with 5–10% CO2. The mixtures were smeared and stained with Giemsa. A minimum of 100 cells were counted in different fields under a microscope at 1000× magnification. The phagocytic assay PA and the phagocytic index (PI) were calculated using the following equations:

$$\textrm{PA}=\left(\textrm{No}.\textrm{of}\ \textrm{ingesting}\ \textrm{phagocytes}/\textrm{total}\ \textrm{No}.\textrm{of}\ \textrm{phagocytes}\right)\times 100$$

$$\textrm{PI}=\textrm{No}.\textrm{of}\ \textrm{ingested}\ \textrm{C}.\textrm{albicans}\ \textrm{cells}/\textrm{No}.\textrm{of}\ \textrm{ingesting}\ \textrm{phagocytes}$$

### Antioxidant and Cytokine Gene Expressions

The impacts of intermittent dietary-active-MOS on the gene expression of immune-related cytokines; interleukin *(IL)-1β,* heat shock *(Hsp)70,* and tumor necrosis factor *(TNF)-α,* besides antioxidant enzymes; superoxide dismutase enzyme (SOD) and catalase (CAT) enzyme. At the end of the trial, three Nile tilapia were used to extract total RNA from the head kidney tissues using the Trizol reagent (iNtRON Biotechnology Inc., Korea) according to the manufacturer’s recommendations.

By Nanodrop D-1000 spectrophotometer, the harvested RNA was evaluated for quantity and quality, spectrophotometer manufactured by NanoDrop Technologies Inc., USA. The obtained cDNA was used as a template in the quantitative real-time PCR assay, and the housekeeping gene was β-actin due to its constitutive gene expression, and the expression was determined using the equation 2^−ΔΔCT^ [55]. All primers are listed in Table 1.

**Table 1:** Sequences of the primers used in the expression of the studied genes

Gene	Primers (5′–3′)	Genbank
*β-actin*	F: CAGGGAGAAGATGACCCAGA R: CAGGGCATAACCCTAGTAGA	EU887951.1
*IL-1β*	F: GACAGCCAAAAGAGGAGC R: TATCAGCGATGGGTGTAG	KF747686.1
*Hsp70*	F: GCTCTGAACCCCAGCAACACT R: TTGTCCTCCCCTTTGTACTCCA	FJ213839.1
*TNF-α*	F: TAGAAGGCAGCGACTCAA R: CCTGGCTGTAGACGAAGT	NM_001279533.1
SOD	F: CATGCCTTCGGAGACAACAC R: ACCTTCTCGTGGATCACCAT	AY491056.1
CAT	F: AGCTCTTCATCCAGAAACGC R: GACGTCAGGCGTCACATCTT	JF801726.1

Note: *IL*, interleukin; *Hsp*, heat shock protein; *TNF*, tumor necrosis factor; *SOD*, superoxide dismutase enzyme; *CAT*, catalase

### DNA Fragmentation Assays

According to Perandones et al. [56], DNA fragmentation was determined in the experimental Nile tilapia. The principle is a bluish color that developed from the reaction of deoxyribose a sugar (derived from DNA) with perchloric acid and the diphenylamine reagent.

### Bacterial Challenge

At the end of the feeding trial, ten Nile tilapia from each group were randomly chosen and injected via intraperitoneally (IP) route with LD_50_ (3 × 10^5^ CFU) of *S. agalactiae* pathogenic strain (accession number OL471408) strain previously isolated and identified by Sherif et al. [57]. In addition, ten fish from the control group were injected with pure saline solution (0.65%) and were considered negative controls [58]. For 14 days, the injected Nile tilapia were kept under observation to calculate the mortality rate (MR %).

$$\textrm{MR}\left(\%\right)=\left(\textrm{number}\ \textrm{of}\ \textrm{deaths}\ \textrm{in}\ \textrm{a}\ \textrm{specific}\ \textrm{period}/\textrm{total}\ \textrm{population}\ \textrm{during}\ \textrm{that}\ \textrm{period}\right)\times 100$$

The relative protection levels (RLP) of SeNPs were evaluated according to Ruangpan et al. [59] as follows:

$$\text{RLP}\%=100\times\left[1-\left(\%\text{deaths}\;\text{in}\;\text{the}\;\text{treated}\;\text{group}/\%\text{deaths}\;\text{in}\;\text{the}\;\text{control}\;\text{group}\right)\right]$$

### Statistics

The obtained data which concerning the impacts of AFB1 contamination and dietary SeNPs in Nile tilapia were analyzed for the significance of differences between the experimental groups at *P* ≤ 0.05 using one-way ANOVA by SPSS version 22 (SPSS Inc., IL, USA). The data are presented as the mean ± standard error.

## Results

### The Characterization of Biological SeNPs

**Fig. 1:**
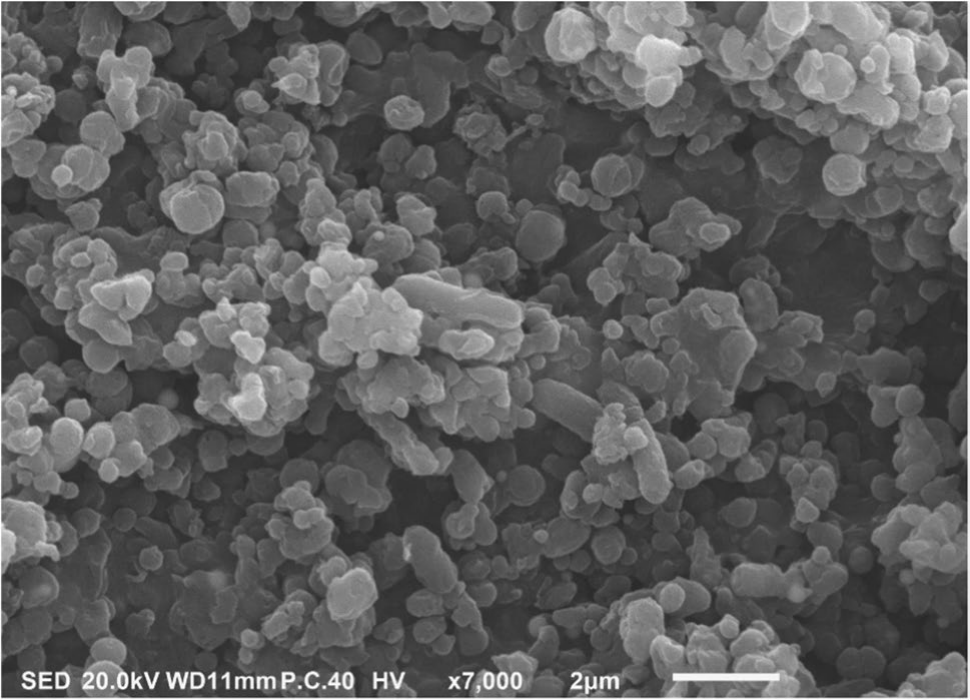
Characterization of SeNPS and *L. bulgaricus* scanned by electron microscope SEM

In Fig. 1, the biological method was used to produce SeNPs with a size ranging between 100 and 200 nm. The final mixture contains lactic acid bacteria *(L. bulgaricus).*

### Activity of Liver Enzymes in Serum of Nile Tilapia

**Fig. 2:**
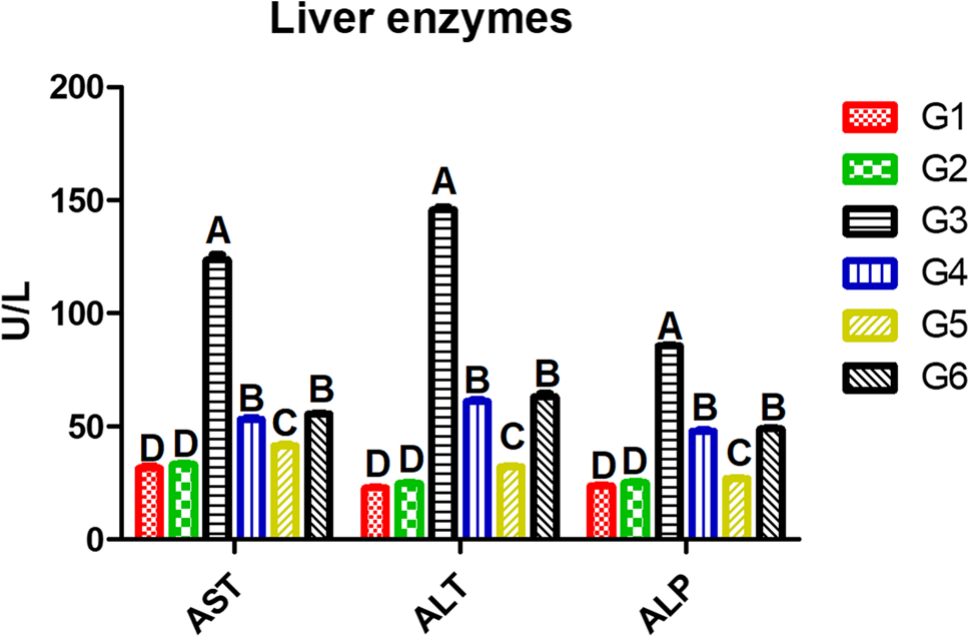
Liver enzymes of the experimental Nile tilapia. Note: AST, aspartate amino transaminase; ALT, alanine amino transaminase; ALP, alkaline phosphatase. Fish groups (G) fed diets: G1, basal diet; G2, SeNP diet; G3, AFB1 diet; G4, AFB1 plus SeNP diet; G5, AFB1 diet then basal diet plus SeNPs; G6, AFB1 diet then AFB1 diet plus SeNPs. Different capital letters indicate significant difference at *P* ≤ 0.05

In Fig. 2, Nile tilapia tested with AFB1-contaminated diets and/or SeNPs, AST, ALT, and ALP levels were upregulated in the serum of fish fed AFB1 diet (G3), 123.5, 145.7, and 85.7, respectively (*P* ≤ 0.05), while fish received SeNPs with free diet after AFB1 diet for 4 weeks (G5) had significantly low levels 41.5, 32.2, and 27 U/L, respectively (*P* ≤ 0.05), compared to G3. Liver enzymes of fish (G4 and G6) that fed AFB1-contaminated diets and received SeNPs were decreased compared to G3.

### Innate Immunity of Nile Tilapia Exposed to AFB1 and Received SeNPs

In Table 2, significant alterations in SAA, OBA, and phagocytic activities (PA and PI) were observed in Nile tilapia fed AFB1 diet (G3), and aflatoxicosis resulted in immunosuppressed status. Supplementation with dietary SeNPs significantly (*P* ≤ 0.05) elevates the immune status (G2) even in fish that received AFB1 diets (G4 and G6).

**Table 2:** Innate immunity of the experimental Nile tilapia

Items	G1	G2	G3	G4	G5	G6
SAA %	33.70**C** ± 0.15	45.20**A** ± 0.85	14.10**E** ± 0.20	25.80**D** ± 0.53	40.60**B** ± 0.70	26.40**D** ± 0.35
OBA (no. of stained cells)	4.33**B** ± 0.70	7.33**A** ± 0.30	1**D** ±0.60	2.70**C** ± 0.30	6.3**A** ± 0.30	1.33**CD** ± 0.30
PA %	35.70**AB** ± 1.20	36.70**A** ± 1.45	20.70**D** ± 0.70	33.30**BC** ± 0.88	37.3A ± 0.30	31**C** ± 1.15
PI (no. of cells)	2.67**A** ± 0.30	3**A** ± 0.60	0.30**B** ± 0.30	1.70**AB** ± 0.67	2.67A ± 0.30	1**B** ± 0.60

Note: *SAA*, serum antibacterial activity; *OBA*, oxidative burst activity; *PA*, phagocytic assay; *PI*, phagocytic index. Fish groups (G) fed diets: *G1*, basal diet; *G2*, SeNP diet; *G3*, AFB1 diet; *G4*, AFB1 plus SeNP diet; *G5*, AFB1 diet then basal diet plus SeNPs; *G6*, AFB1 diet then AFB1 diet plus SeNPs. Different capital letters within the same row indicate significant difference at *P* ≤ 0.05

Dietary SeNPs boost the antibacterial compound in fish serum SAA was significantly raised (45.20%) compared to the control 33.70% (*P* ≤ 0.05); meanwhile, a drastic decline was observed in groups exposed to AFB1 (G3, 14.10%) and SeNP supplementation could partially restore SAA (G5, 40.60%).

The ability of phagocytic cells to adhere to (OBA) and phagocyte (PA and PI) microbes was significantly restored after ceasing AFB1 exposure and adding dietary SeNPs (G5) (*P* ≤ 0.05).

### Gene Expression of Antioxidant Enzymes in the Liver of Nile Tilapia Exposed to AFB1 and Received SeNPs

**Fig. 3:**
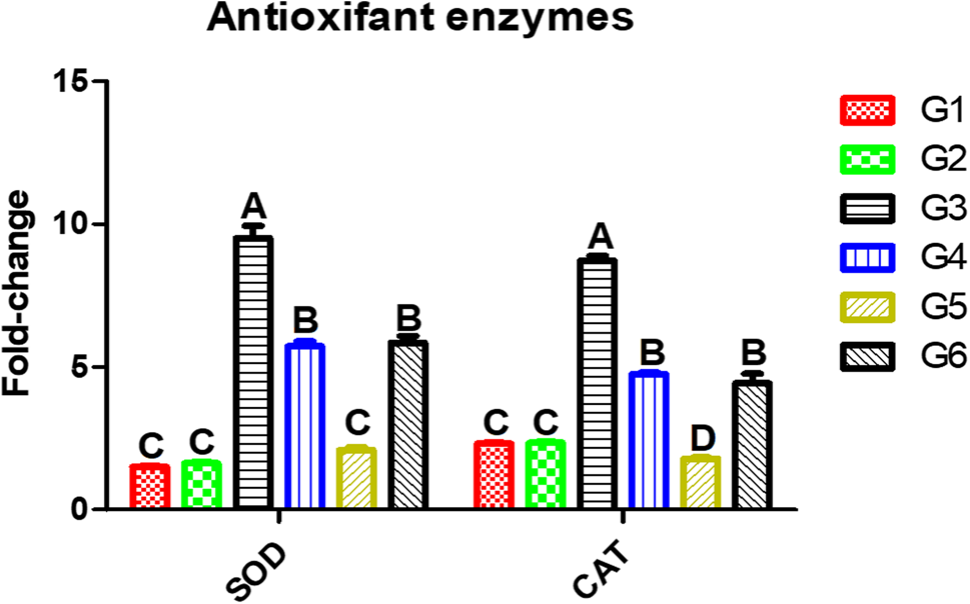
Gene expression antioxidant enzymes of the experimental Nile tilapia. Note: SOD, superoxide dismutase enzyme; CAT, catalase. Fish groups (G) fed diets: G1, basal diet; G2, SeNP diet; G3, AFB1 diet; G4, AFB1 plus SeNP diet; G5, AFB1 diet then basal diet plus SeNPs; G6, AFB1 diet then AFB1 diet plus SeNPs. Different capital letters indicate significant difference at *P* ≤ 0.05

In Fig. 3, in Nile tilapia, gene expressions of SOD and CAT were significantly increased in response to aflatoxicosis (G3) 9.5- and 8.72-fold change, respectively (*P* ≤ 0.05) compared to the other groups; meanwhile, ceasing AFB1 diet and feeding dietary SeNPs (G5) could counteract the generated oxidative status and downregulate the gene expressions of antioxidant enzymes 2.1- and 1.8-fold change, respectively.

### Gene Expression of Cytokines in the Liver of Nile Tilapia Exposed to AFB1 and Received SeNPs

**Fig. 4:**
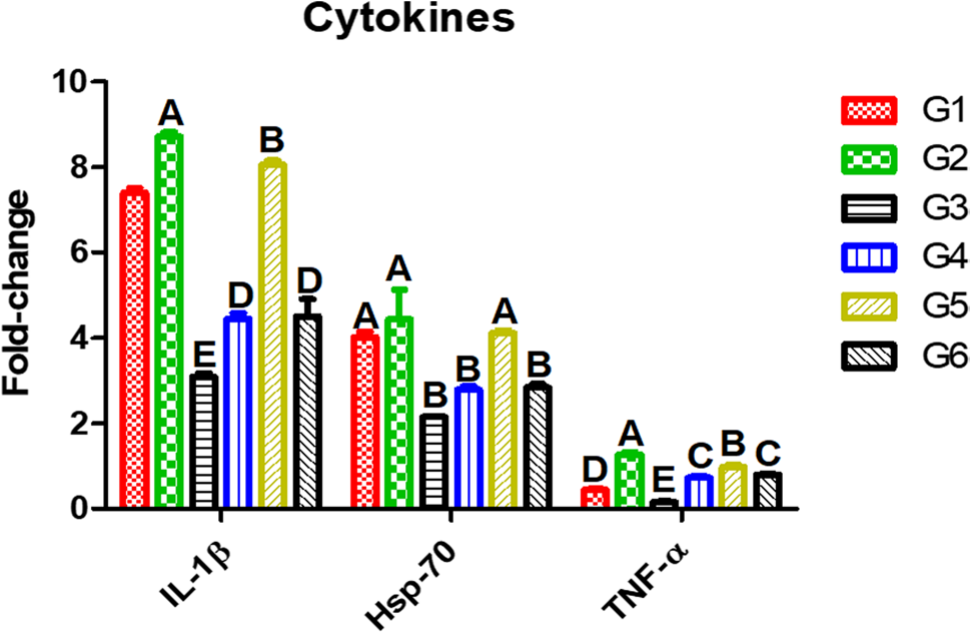
Gene expression of cytokines of the experimental Nile tilapia. IL, interleukin; Hsp, heat shock protein; TNF, tumor necrosis factor. Fish groups (G) fed diets: G1, basal diet; G2, SeNP diet; G3, AFB1 diet; G4, AFB1 plus SeNP diet; G5, AFB1 diet then basal diet plus SeNPs; G6, AFB1 diet then AFB1 diet plus SeNPs. Different capital letters indicate significant difference at *P* ≤ 0.05

In Fig. 4, to study the impacts of AFB1 on the immune system, the gene expressions of *IL-1β*, *Hsp70*, and *TNF-α* were assessed in experimental Nile tilapia. Regardless of SeNP addition, fish received AFB1 diets were immunosuppressed as *IL-1β*, *Hsp70*, and *TNF-α* were significantly decreased (*P* ≤ 0.05), while Nile tilapia received dietary SeNPs were significantly immune enhanced (*P* ≤ 0.05).

### DNA Fragmentation of Liver Cells of Nile Tilapia Exposed to AFB1 and Received SeNPs

**Fig. 5:**
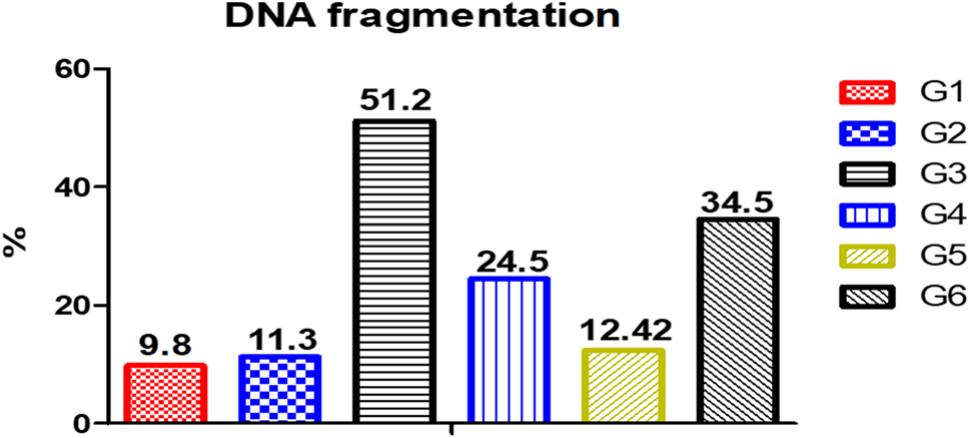
DNA fragmentation of the experimental Nile tilapia. IL, interleukin; Hsp, heat shock protein; TNF, tumor necrosis factor. Fish groups (G) fed diets: G1, basal diet; G2, SeNP diet; G3, AFB1 diet; G4, AFB1 plus SeNP diet; G5, AFB1 diet then basal diet plus SeNPs; G6, AFB1 diet then AFB1 diet plus SeNPs

In Fig. 5, feeding an AFB1-contaminated diet increased DNA fragmentation (G3) reaching about 51.2% compared to the control 9.8%. The addition of SeNPs in Nile tilapia diets could ameliorate the withdrawals of aflatoxicosis and decrease DNA fragmentation to 12.42%, 24.5%, and 34.5% in G5, G4, and G6, respectively.

### Experimental Infection Test

In Table 3, Nile tilapia fed dietary SeNPs and experimentally infected with *S. agalactiae* had a low MR of 40% providing 33.3% of RLP. Fish feed contaminated diets supplemented with SeNPs had a similar MR% as the control ones about 60%, while the MR of fish that received SeNPs with free diet (G5) was similar to G2 at 40%. As RPL% was calculated, fish of G3 were considered the control positive to those fed contaminated diets and received SeNPs (G4, G5, and G6) providing 40%, 60%, and 40%, respectively.

**Table 3:** The fish-related deaths of the experimental Nile tilapia and relative level of protection of SeNPs

Items	Free diet			Contaminated diet			
	NC	G1 (CP)	G2	G3 (CP)	G4	G5	G6
Fish no.	10	10	10	10	10	10	10
Fish death	2	6	4	10	6	4	6
MR%	20	60	40	100	60	40	60
RLP%	**-**	**-**	**33.3**	**-**	**40**	**60**	**40**

Note: Fish groups (G) fed diets: *G1*, basal diet; *G2*, SeNP diet; *G3*, AFB1 diet; *G4*, AFB1 plus SeNP diet; *G5*, AFB1 diet then basal diet plus SeNPs; *G6*, AFB1 diet then AFB1 diet plus SeNPs. *NC*, negative control; *CP*, control positive

## Discussion

Aflatoxins adversely affected the aquaculture industry such as the cost of toxin elimination chelating materials and the loss in net fish production. These toxins induce oxidative inflammation by generating ROS production, causing cellular damage. Selenium is a well-known antioxidant agent that could be used as feed supplementation for aquatic animals.

In this study, the biologically manufactured SeNPs with 100- to 200-nm size were used in a feeding trial at a concentration of 1 mg/kg fish feed to boost the immunity of Nile tilapia and to mitigate oxidative stress which was induced by aflatoxicosis. Different dietary requirements were set for Se, with a maximum of 0.2 mg/kg in animal feed [60], between 0.18 and 0.38 mg/kg [61], 1.06 mg/kg and 2.06 mg/kg Se for Nile tilapia [23]. In this experiment, no deaths were recorded in the experimentally fed contaminated diet with AFB1 and/or SeNPs. Accordingly, biological-origin SeNPs were used safely in animal feed [20]. Similarly, SeNPs as its toxicity less by 3.6 times than selenomethionine [62]. In accordance, Gonçalves et al. [63] observed a high survival rate of 99.0–99.7% in Tra catfish *(Pangasius hypophthalmus)* after 12 weeks of exposure to AFB1 (0, 50, 100, and 250 μg/kg), whereas Nile tilapia fed with 2000 μg/kg of AFB1 had a survival rate of only 82.2% [64].

Before macroscopic changes took place, alterations in serum AST and ALT could be noticed after receiving dietary AFB1 in gibel carp [65] and Tra catfish [63]**.** In this study, Nile tilapia that fed AFB1-contaminated diets, AST, ALT, and ALP levels were upregulated in the serum at 123.5, 145.7, and 85.7 U/L, respectively. Similarly, AST and ALT were increased in the serum of Nile tilapia that received dietary AFB1 at a concentration of 20 and 100 μg/kg for 12 weeks [2]. These findings could be explained by the findings of [66] who stated that large amounts of toxins metabolized in the liver that damage the tissue and raise the enzyme activities. Other results found by Deng et al. [67]; ALT and AST levels did not alter in Nile tilapia fed AFB1-contaminated diet at a concentration of 1.641 μg/kg and in Gibel carps at a concentration up to 1000 μg/kg. On the contrary, some researchers found that fish fed dietary AFB1 at a concentration of 100 μg/kg fish feed did not affect such as Nile tilapia [64, 68] and South American catfish *(Rhamdia quelen)* [69]. These different findings related to AFB1 concentration, feeding period, and fish species.

No changes were recorded in serum AST, ALT, and ALP levels of the experimental fish that received dietary SeNPs. Similarly, Liu et al. [70] found that ALT and AST levels in the serum of grass carp (*Ctenopharyngodon Idella*) were significantly increased in fish fed with a high-fat diet and dietary SeNPs restored normal levels. Meanwhile, Neamat-Allah et al. [71] stated that ALT, AST, ALP, LDH, and creatinine in Nile tilapia were significantly increased in serum with dietary SeNPs. This could be due to the liver of fish being the site of Se metabolism and accumulation [72]. These authors use high dietary SeNPs and/or longer periods; in addition, the raise was only in numbers not in duplicates of the control level that could not considered clinical cases.

Aflatoxins are immunosuppressive substances that cause weakness the innate immunity [73]. Experimental Nile tilapia fed an AFB1-contaminated diet were immunosuppressed, with SAA, OBA, and phagocytic activities significantly declined. Accordingly, aflatoxin metabolites in the liver could bind with cellular macromolecules preventing their synthesis [74] and leading to a significant decline of serum proteins in sea bass *(Dicentrarchus labrax)* [75] and Nile tilapia **[**76**].** Dietary-SeNPs enhanced the innate immunity of Nile tilapia. In accordance, Se is a part of selenoprotein that could increase serum protein production, so using SeNPs as a dietary supplementation could enhance non-specific immunity [77, 78]; in addition, they were less toxic to immune cells compared with organic and inorganic forms [79].

The gene expression of SOD and CAT were significantly downregulated in the experimental Nile tilapia fed AFB1 diet compared to the control fish. Similarly, the activities of CAT and GSH were decreased leading to a decline of antioxidant capacity in Nile tilapia that fed diets contaminated with AFB1 [64]. The study findings indicated that Se supplementation could ameliorate the generated oxidative stress resulting from AFB1. Glutathione peroxidase (GPx), CAT, SOD, myeloperoxidase (MPO), and lysozyme (LZM) in Nile tilapia were improved by dietary Se supplementation [80, 81]. In addition, oxidative stress could be significantly mitigated by using dietary SeNPs that stimulate the formation of glutathione [6, 82, 83].

From the obtained results, Nile tilapia that fed an AFB1-contaminated diet showed an upsurge of DNA fragmentation in hepatic tissue [84], and dietary SeNPs could counteract these withdrawals. Similarly, SeNPs overcome the adverse impacts of aflatoxicosis by increasing the synthesis of protein and DNA reducing the deaths of lymphocyte cells and DNA fragmentation [85], also suppressing the pro-apoptotic proteins leading to a decrease of oxidative damage in the liver [33], along with triggering the carotenoids and vitamin A remediation role [83].

The gene expressions of *IL-1β*, *Hsp-70*, and *TNF-α* of the experimental Nile tilapia were decreased after receiving AFB1-contaminated feed while dietary SeNPs could ameliorate such hazards. In accordance, feeding AFB1-contaminated diets resulted in heightened expression of *IL-6*, *IFN-γ*, and *IL-10*; impaired lymphocyte; and delayed cell-mediated immune response [86], while Se deficiency aggravated AFB1-induced immunotoxicity [87]. Similarly, dietary SeNPs (5 mg/kg) protected rainbow trout (*Oncorhynchus mykiss*) against oxidative stress by inducing the gene expression of *Hsp70b*, *Hsp90α*, and *Hsp30*, selenoproteins (*Gpx1a*, *Gpx1b1*, and *Trx*), and *TGF-β* as well as catalase activity [88].

The experimental Nile tilapia were experimentally infected with *S. agalactiae;* high MR% was recorded in fish that fed AFB1-contaminated feed while dietary SeNPs provided high RPL%. Similarly, survival rates of Nile tilapia were upsurged by dietary Se when they were challenged against *S. agalactiae* infection [81, 89], *Aeromonas hydrophila* [90, 91], and *S. iniae* [71]**.** In addition, antibacterial effects were increased by increasing Se concentration as 0.1–0.3 mg/kg exerting more activity than 1.5 mg/kg, this is due to the peptidoglycan layer destruction in the bacterial cell membrane [92] that kills the pathogen via cytoplasmic leakage [93]. Similarly, Nile tilapia that fed a diet supplemented with PSP-SeNPs (selenium nanoparticles coated with polysaccharide-protein) at a rate of 0.1–0.3 mg/kg could combat oxidative stress and experimental *Streptococcus agalactiae* infection [92]. Accordingly, with different forms of Se, Hassan et al. [94] found that organic selenium could ameliorate oxidative stress following the exposure to single exposure to malathion and glyphosate, as well as combat *A. hydrophila* infection.

## Conclusion

From previous findings, SeNPs could partially ameliorate aflatoxicosis (AFB1) in Nile tilapia. Oxidative stress was the most prominent sign accompanied by AFB1 and demonstrated by increasing DNA fragmentation and gene expressions of antioxidant enzymes along with immunosuppression. The addition of SeNPs enhanced the antioxidant status, especially with a free AFB1 diet. Meanwhile, continuous feeding on contaminated diets with/without SeNP supplementation fish could not restore normal physiological parameters. Nile tilapia that received AFB1 diet became more vulnerable to bacterial infection while SeNP supplementation provided influential protection.

### Supplementary information

ESM 1 (JPG 142 kb)
