# Supplementary figures and images for: Selenium Nanoparticles Ameliorate Adverse Impacts of Aflatoxin in Nile Tilapia with Special Reference to Streptococcus agalactiae Infection
Source: Biol Trace Elem Res. 2023 Dec 26;202(10):4767–77. doi: 10.1007/s12011-023-04031-1 (PMC11339097; doi:10.1007/s12011-023-04031-1)

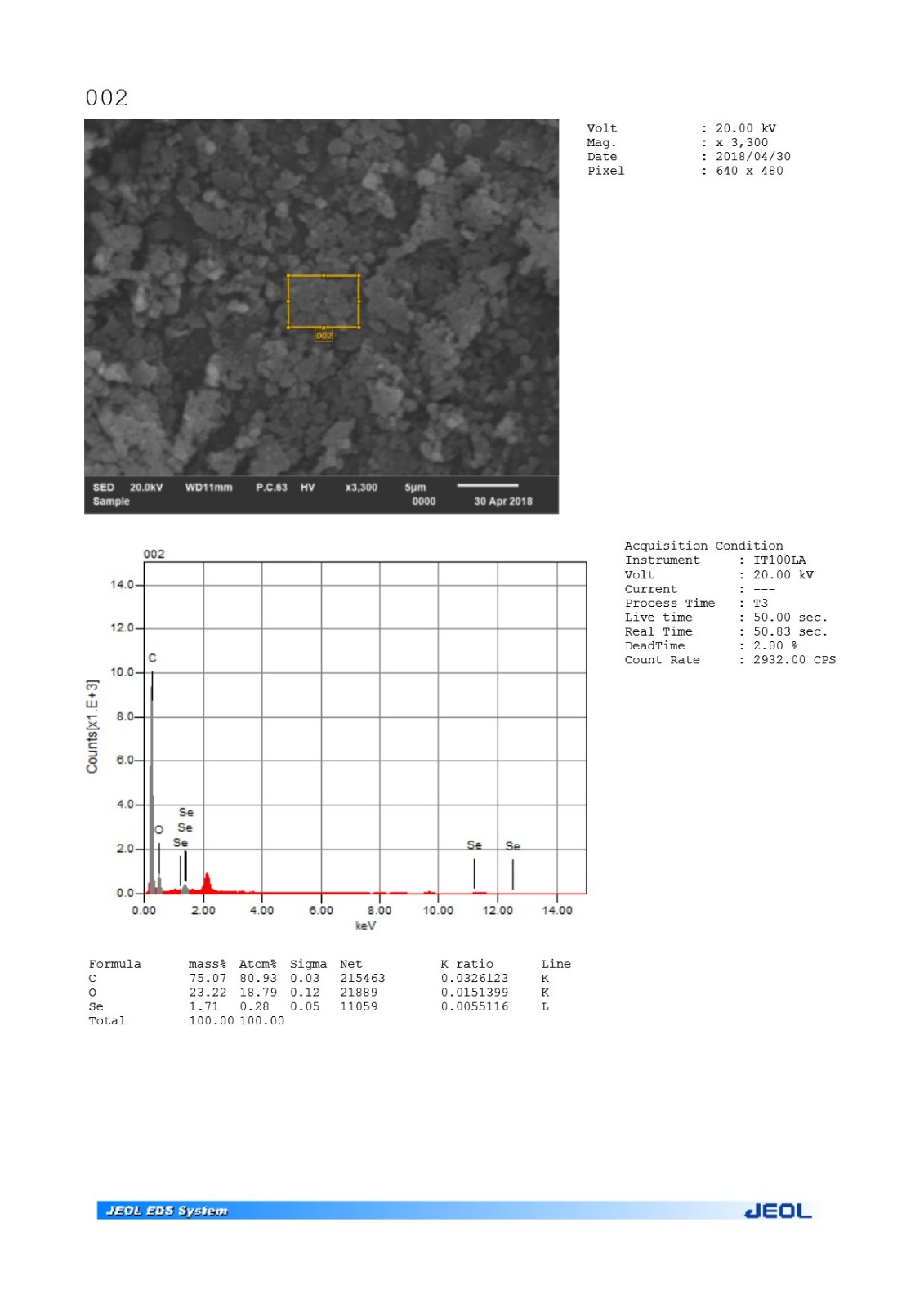

Supplement: Supplementary file 1 — (JPG 142 kb) [file 12011_2023_4031_MOESM1_ESM.jpg]
